# Landscape‐Scale Effects of Season and Predation Risk on the Terrestrial Behavior of Chacma Baboons (*Papio ursinus*)

**DOI:** 10.1002/ajpa.70052

**Published:** 2025-04-15

**Authors:** Philippa Hammond, Kaitlyn Gaynor, Tara Easter, Dora Biro, Susana Carvalho

**Affiliations:** ^1^ Primate Models for Behavioural Evolution Lab, School of Anthropology and Museum Ethnography University of Oxford Oxford UK; ^2^ Departments of Zoology and Botany University of British Columbia Vancouver British Columbia Canada; ^3^ School for Environment and Sustainability University of Michigan Ann Arbor Michigan USA; ^4^ Department of Zoology University of Oxford Oxford UK; ^5^ Department of Brain and Cognitive Sciences University of Rochester Rochester New York USA; ^6^ Paleo‐Primate Project, Gorongosa National Park Chitengo Mozambique; ^7^ Interdisciplinary Center for Archaeology and Evolution of Human Behaviour (ICArEHB) Universidade Do Algarve Faro Portugal

**Keywords:** baboon, diel activity patterns, leopard, predation risk, terrestriality

## Abstract

**Objectives:**

“Terrestrial” primates are not common nor well defined across the order. In those species that do use the ground, terrestriality is rarely documented outside daylight hours. Predation risk is thought to have shaped conserved behaviors like primates' selection of arboreal sleep sites, but it is less clear—particularly at the landscape scale—how predation risk interacts with other ecological and seasonal variables to drive terrestriality. This camera trapping study investigates patterns in terrestrial behavior both spatially and temporally across neighboring populations of chacma baboons.

**Materials and Methods:**

We use camera trap data from two terrestrial grids, one established within and one outside the boundaries of Gorongosa National Park, Mozambique. We model how baboon terrestrial activity varies with woody cover, proximity to water, season, anthropogenic variables, as well as predation risk. We also model how terrestrial activity varies across the diel cycle and use overlap analyses to explore differences in the baboon populations' activity patterns.

**Results:**

We find no significant predictors of geospatial variation in the terrestrial activity of baboons across each grid but do find evidence of higher terrestrial activity in the late dry season. We also find significantly different diel patterns of baboon activity detected across each grid.

**Discussion:**

Baboons likely use the ground more in the dry season for accessing water and resources when arboreal foods are less abundant. Diel variation between the two populations suggests that baboons might utilize the ground more during “riskier” crepuscular and nocturnal hours where leopards are not present.

**Summary**

- Baboon terrestrial activity varies across seasonal and diel cycles.
- Baboons use the ground more in the dry season, but avoid terrestriality at riskier times of day, particularly in a landscape where their primary predator is present.

## 1 Introduction

Most primate species spend their time almost exclusively in the trees. By one estimate, terrestriality is seen in less than 6% of extant primate species, with semi‐terrestriality reported in an additional 9% of species (Galán‐Acedo et al. 2019). However, there are no clear definitions, nor standardized methods for classifying “terrestrial” and “semi‐terrestrial” primates. For example, the estimates above are taken from a database where species described as “carrying out most of their daily activity on the ground” were labeled as terrestrial, and those “commonly active on both substrates” were labeled as “both terrestrial and arboreal”, but the data come from studies and sites across the world that all use different metrics. The classification might also depend upon researchers' behaviors of interest. Chimpanzees ( *Pan troglodytes* ), for example, often travel, rest, and socialize on the ground, but do much of their feeding in trees (Hunt 2016). Even species like baboons (genus *Papio*), described as the “most terrestrial” of non‐human primates, still sleep in trees (J. R. Anderson 1998).

Indeed, baboons' use of the ground is confined almost entirely to daylight hours as they reliably return to trees or cliffs to sleep every night (Altmann and Altmann 1970; Bidner et al. 2018; Cowlishaw 1994; Hamilton 1982; Markham et al. 2016). Both their diurnal activity patterns and sleep site preferences have been attributed to the strong selective force of predation risk, which is highest at night when large terrestrial carnivores are more active and when the probability of detection or escape are lower due to reduced visibility (Altmann and Altmann 1970; C. M. Anderson 1986; Bidner et al. 2018; Cowlishaw 1994; Hamilton 1982). While sleeping above the ground appears to be a fixed trait in baboons—even among those in captivity who are safe from predation (Samson and Shumaker 2015) – there is evidence from several primate species that frequency of diurnal terrestrial behavior is affected by the level of risk perceived in the environment (Campbell et al. 2005; Hammond et al. 2022; Mourthé et al. 2007; Nowak et al. 2014; Souza‐Alves et al. 2019). Many of these studies focus on primate species who rarely descend to the ground, and most explore immediate effects of perceived risk on substrate choice or types of terrestrial behavior. However, the longer‐term effects of risk on terrestriality are less clear, particularly in relation to how they interact with spatial variables such as the availability of trees, prevalence and distribution of water, and anthropogenic disturbance (human activity) in the environment, as well as temporal variables such as seasonal and diel cycles, all of which are likely to be key drivers of ground use in terrestrial primate species. This study provides a systematic landscape‐scale investigation of the effects of such variables on baboons' terrestrial activity patterns.

There is growing recognition that “the landscape of fear”—perceived spatial variation in predation risk across a landscape—is not a static representation held by animals, but varies with factors like habitat‐structure, season, and time of day, as well as the diversity and hunting styles of all predators in the environment (Gaynor et al. 2019; Kohl et al. 2018; Palmer et al. 2017). Interactions among these dynamic variables are thought to shift animals' perceptions of risk across both space and time, influencing a range of their behaviors, from immediate reactive anti‐predation strategies such as vigilance, to adaptations such as diurnal activity patterns or group living (Palmer et al. 2017; Palmer and Packer 2021; Tambling et al. 2015; Willems and Hill 2009; Winnie and Creel 2017). Monitoring these dynamics at a landscape scale is now possible thanks to developments in remote‐sensing methods such as bio‐logging, camera trapping, and extraction of ecological data from satellite imagery. Remote‐sensing technologies facilitate synchronous monitoring of multiple species within an environment, alongside the collection of relevant ecological variables, all while minimizing human interference and improving opportunities for standardization across long‐term and cross‐site studies (Pardo et al. 2021; Rowcliffe 2017).

These remote‐sensing methods are increasingly being adopted in primatology, which has long relied on observational studies where researchers follow one or several primate groups within a landscape, recording the behaviors of individuals within each group (Janson 2012). While this immersive strategy has provided great insights into primate behaviors and social interactions, it is difficult and costly to simultaneously monitor more than a handful of neighboring primate troops at best. Additionally, the suitability of observational methods varies by the topic of investigation. For example, data on predation risk have often been derived from primate–predator encounters that occur while researchers are in the field. Such encounters are limited, both by the fact that many large carnivores are most active at night when researchers are usually not present, and because human presence can actually deter predators from the primates under observation (Isbell and Young 1993; Nowak et al. 2014).

Recent remote‐sensing studies provide insights into more nuanced effects of predation risk on primate behavior. For example, a study that used GPS collars to simultaneously track leopards ( *Panthera pardus* ), vervet monkeys ( *Chlorocebus pygerythrus* ), and olive baboons ( *Papio anubis* ) found that the leopards appeared to target the two primate species at different times of day. Leopards preferentially hunted the smaller‐bodied vervets during the day while actively avoiding baboons, who are known to attack and even kill leopards (Cowlishaw 1994; Crofoot 2013). However, the leopards then targeted the larger‐bodied baboons (whose size falls within leopards' preferred prey size) at night, when detection and pre‐emptive attack by the baboons were less likely (Isbell et al. 2018). This highlights how risk from a particular predator can change across the diel period, and why baboons' nocturnal activities might be particularly affected by the presence of leopards. Indeed, collar data from the same field site showed that only 5% of all departures from sleep sites occurred before sunrise and there was evidence that baboons left their riverine sleep sites significantly later on mornings after a leopard had visited the site (Bidner et al. 2018). Similarly, a camera trapping study found temporal partitioning between leopards and several of their prey species, with yellow baboon ( *Papio cynocephalus* ) activity showing the least degree of overlap with leopard activity (Havmøller et al. 2020). It is perhaps because primates can evade predators along two dimensions (avoiding them in the landscape and ascending into trees) that terrestrial camera traps capture this signal of clearer temporal partitioning by baboons than by all the ungulate prey species at the site.

The research findings described above not only highlight the temporal dynamics of landscapes of fear, but also the potential significance of predator species and hunting style on prey behavior. Leopards, whose preferred prey size ranges between 10 and 40 kg, are considered to be baboons' primary predator in areas where they occur (Hayward et al. 2006; Zuberbühler and Jenny 2002). Meanwhile, lions generally prefer larger‐bodied prey—between 190 and 550 kgs—although they do sometimes kill baboons (Busse 1980; Hayward and Kerley 2005). In this study, we investigate the effects of spatial variation in predator presence on baboons' terrestrial activity across the landscape, and explore whether baboons are more likely to avoid leopards than lions (either spatially or temporally), based on the hypothesis that leopards pose a greater risk to baboons than lions.

To explore these questions, we used terrestrial camera trap data from two neighboring populations of chacma baboons ( *Papio ursinus* ) in Mozambique, one of which was exposed to the risk of predation by lions ( *Panthera leo* ), and the other to the risk of predation by leopards ( *Panthera pardus* ). First, we tested whether spatial variation in predator presence affected the frequency of terrestrial activity in either of the baboon populations, hypothesizing that baboons would be less active on the ground in areas where predators were most active. Alongside this indicator of localized risk, we tested how baboon activity varied with woody cover (as an indicator of tree availability), water availability, seasonality, and anthropogenic features across each landscape. We assumed that the availability of trees in any environment is a fundamental driver of terrestrial activity in baboons, and that the proximity of water on the ground (in rivers, lakes, or pans) might increase ground use. We predicted that seasonality would also affect terrestrial activity, with baboons likely descending to the ground more frequently in the dry season to travel to scarce water sources and to utilize fallback foods like grasses and tubers when arboreal fruits are less abundant. Anthropogenic features of the environment might also impact the detection of baboon activity and were therefore also considered in our analyses. For example, baboons might be attracted to human settlements for foraging opportunities, or they might avoid human disturbance if they are unhabituated to people. Research from other study sites also indicates that baboons preferentially utilize roads for travel (Kiffner et al. 2022; Strandburg‐Peshkin et al. 2017), which might lead to higher detection rates of the species closer to roads.

As well as exploring how baboon activity varies spatially and seasonally, in this study we compare the daily activity patterns of baboons across each of the camera trap grids to investigate their temporal partitioning with two different apex predators. Based on the hypothesis that baboons perceive a higher risk of leopards than lions, and that crepuscular and nocturnal hours are “riskier” times of day than daylight hours, we test (a) the extent to which the two baboon populations show temporal partitioning with the respective predator species in their environments, (b) the extent to which the two baboon populations' activity patterns overlap, and (c) whether the species of predator affects the times of day during which baboons are most often active on the ground.

## 2 Methods

### 2.1 Study Site

Gorongosa National Park (GNP), Mozambique (−18.96°, 34.36°), is composed of ca. 4000 km^2^ of heterogeneous habitats and is inhabited by over 200 troops of chacma baboons (troop sizes range from < 10 to > 150 individuals), among many other species (Stalmans et al. 2018). While apex predators including lions, leopards, wild dogs ( *Lycaon pictus* ) and spotted hyenas ( *Crocuta crocuta* ) used to be widespread in the park (Tinley 1977), civil war in Mozambique between 1977 and 1992 decimated wildlife in the park, and apex predators were almost completely lost from the landscape (Daskin et al. 2016; Stalmans et al. 2019), except for a small but now growing lion population (Bouley et al. 2018). Since 2018, both wild dogs and leopards have been successfully reintroduced to the park, but these reintroductions occurred after the data collection period for this study (Bouley et al. 2021).

Situated at the southern end of the East African Rift System, a 40‐km‐wide valley runs through GNP, with a grassland floodplain and Lake Urema at its center. Woodland savannas surround the floodplain, predominantly composed of *Acacia‐Combretum* assemblages, with some palmveld and closed‐canopy forest. The area has a sub‐tropical climate, with the majority of rainfall occurring between December and March. In the valley, typical annual rainfall is 700–900 mm, but it is higher on the surrounding plateaus. South of Lake Urema, there is a road network used by a small but growing tourist operation, as well as researchers and rangers (Gaynor et al. 2021; Stalmans and Beilfuss 2008).

A 5333‐km^2^ buffer zone surrounds GNP and is designated as a mixed‐use area inhabited by 200,000 subsistence farmers. To the east of Gorongosa's park boundaries and buffer zone lies a 460‐km^2^ forestry concession (FC), certified by the Forest Stewardship Council for low intensity, rotational timber harvesting (Easter et al. 2019). The area is composed of predominantly tall miombo woodlands, with some variation in vegetation density between more open grasslands and closed‐canopy riparian areas, but generally tree coverage is more homogenous than within the park's boundaries. The concession is inhabited by several mammalian species, with leopards as the only known apex predator in the area. The concession partly contains a human settlement, as well as several households and a sawmill. There is a simple road network within the concession used mainly for harvesting timber and to connect the sawmill and community with main roads (Easter et al. 2019).

### 2.2 Camera Trap Grids

**FIGURE 1:**
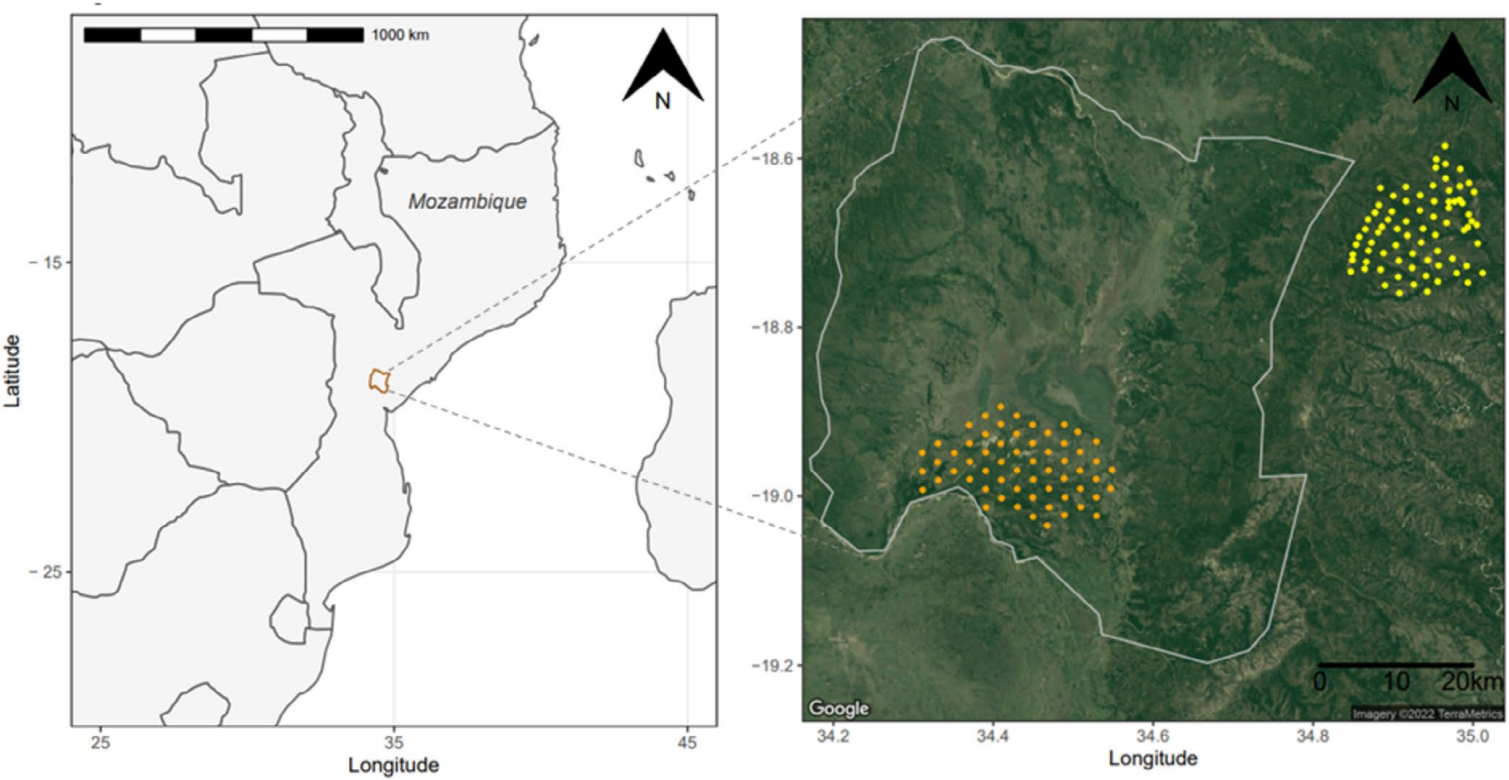
Map showing the position of Gorongosa National Park (GNP) in Mozambique (on left) and the positions of two camera trap grids in relation to the park's boundaries (on right). The position of each camera is marked in orange for the GNP grid and in yellow for the forestry concession (FC) grid.

This study draws on data collected from two camera trap grids, mapped in Figure 1. The GNP grid was established in 2016, covering approximately 300 km^2^ within the park's main boundaries (Gaynor et al. 2021). The grid is part of ongoing research, but data analyzed in this study were collected between June 2016 and September 2018. Tree coverage and habitat type vary considerably across the GNP grid area, as does each camera's distance from roads and water sources. Thus, the grid facilitates exploration of how baboon activity varies with these ecological variables, as well as by season. Lions were the only apex predator inhabiting the gridded area during the study period.

The second camera trap grid was established within the sustainable‐use FC east of the park's boundaries (Easter et al. 2019). The FC grid operated between June and October 2017 and had a slightly different format than the GNP grid (see Table 1). The area covered by the FC grid is characterized by denser and more homogenous tree coverage than the area covered by the GNP grid, and the only known apex predators in the area are leopards.

**TABLE 1:** Operating periods, procedures, and features of the GNP and FC camera trap grids.

	Gorongosa grid	FC grid
Operating days		
June 2016–Sept 2018	26,523	NA
Mean (min–max) per site	442 (68–789)	NA
June–Oct 2017	5319	2090
Mean (min–max) per site	89 (0–129)	28 (18–58)
Total area monitored by grid	300 km^2^	320 km^2^
Total camera trap sites	60	75
Hexagonal grid cell size	5 km^2^	4 km^2^
Distance between sites	Approx. 2.4 km	Approx. 2 km
Operating procedure	Bushnell Trophy Cams, single camera per site, mounted throughout the operating period, active 24 h a day. All cameras placed roughly at center of the grid cell. Checked every 3 months.	Bushnell Trophy Cams, paired cameras at most sites, rotated in four successive blocks, active 24 h a day. Twenty‐two cameras specifically placed along roads/game trails. Checked monthly.
Photo capture procedure	Bursts of two with a 30‐s delay, independent detections classified using a 30‐min interval.	Bursts of two with a 2‐s delay, independent detections classified using a 30‐min interval.

Both grids were established with camera trap sites placed roughly at the center of evenly spaced hexagonal grid cells, although the FC grid also included some cameras specifically placed along roads. The GNP grid used a single camera trap at each site, while most FC sites had paired cameras (to aid in identifying individual leopards). This difference in setup is not expected to influence results, given that baboons' large group sizes give them a high probability of detection (Gaynor et al. 2021). Cameras were mounted on trees, approximately a meter off the ground, and were motion‐triggered to take two photographs per trigger (within < 1 s), classified as a single detection. For paired‐camera sites, animals captured by both cameras at the same time were also classified as a single detection. The grids' features and operating procedures are summarized in Table 1.

### 2.3 Data Processing

The species captured in images across both grids were identified and labeled manually by researchers and assistants. The R package “camtrapR” (Niedballa et al. 2016) was used to generate a timestamped record of all species detections across both grids. The records from each grid were then cleaned to generate data sets of independent species detections, classified using a 30‐min interval between detections of the same species at the same site. While somewhat arbitrary, this is a standard independence time interval used in camera trapping research, and we consider it long enough to account for baboons' average group size and travel speed (Sollmann 2018).

The GPS coordinates for each camera station in each grid were recorded alongside several ecological variables when the grids were first established. These include each camera's distance from the nearest river, nearest road, and nearest human settlement. For the GNP grid, there is also a measure of each camera's distance from Lake Urema, which is the largest permanent water source in the park. To these variables, we added an estimate of “woody cover” at each camera site, under the assumption that the availability of trees would explain some variation in baboons' terrestrial activity. Using the cameras' GPS points, we extracted this estimate from an existing database of woody cover across Sub‐Saharan Africa at a resolution of 1 km (Hanan et al. 2020). While this metric includes both trees and shrubs in its estimate of woody cover, and is thus imprecise, we considered it more applicable than a measure like NDVI, which might not distinguish between trees and grasses (Madonsela et al. 2018; Martinez and Labib 2023).

While the FC grid only operated during the dry season, the GNP grid captured data across the annual cycle and all species detections were therefore classified as “wet” (December–March), “early dry” (April–July), or “late dry” (August–November). For analyses of activity across different diel periods, species detections were classified as “Dawn” (the 60 min before and 60 min after sunrise), “Dusk” (the 60 min before and 60 min after sunset), “Day” (the time between dawn and dusk periods), and “Night” (the time between dusk and dawn periods). Sunrise and sunset times for Gorongosa were identified using the website: https://www.timeanddate.com/sun/ and all timestamped images were classified according to the diel period in which they were captured.

Predator presence across each grid was classified using a binary system; camera sites at which lions or leopards were detected at least once during the study period were labeled as having localized predator activity (“Predators”), while all other cameras were labeled as having no localized predator activity (“No predators”). Of course, the absence of predator detections at a camera trap site does not mean that there are no predators active in that area of the landscape. However, this binary classification system serves as a proxy that aims to capture the areas in which predators were most active.

### 2.4 Statistical Analyses

#### 2.4.1 Spatial and Seasonal Variation in Baboon Activity: Does Terrestrial Activity Vary With Localized Predator Activity, Ecological, Seasonal, and Anthropogenic Variables?

We ran two sets of analyses to explore how baboons' terrestriality was influenced by spatial variation in predator activity, as well as other ecological and seasonal variables. For both sets of analyses, baboon activity was quantified using a Relative Activity Index (RAI), which represents the number of independent baboon detections captured per 100 operating hours at each camera site. In the first set of analyses, we ran generalized linear mixed‐effects models (GLMMs), using only data from the GNP grid so that seasonality could be included in the model. Camera site was thus included as a random variable to account for repeated sampling of each site across seasons. The ecological variables included in the models were woody cover, distance from the nearest river, distance from Lake Urema, season, and predator presence. Under the assumption that anthropogenic activity in the environment might also have some effect on baboon activity, we ran a second model that included all ecological variables plus distance from the nearest road and distance from the nearest human settlement. All continuous covariates were centered and scaled prior to modeling, and the log‐transformed value of operating hours per camera per season was included as an offset term. We calculated and compared the Akaike information criterion (AIC) scores for each of these two models, taking the model with the lowest AIC score as the model that provides the best explanation of the variation in the data.

For the second set of analyses, we included data from both the GNP and FC grids, limiting our analysis to a single dry season period when both grids were active. For these analyses, we fitted negative binomial generalized linear models to the data to account for zero inflation in the data. The ecological fixed variables included in the model were woody cover, distance from the nearest water source (lake for GNP cameras and river for FC cameras), and predator presence. Location (“GNP” or “FC”) was also included as a fixed variable. Again, the log‐transformed value of operating hours per camera was included as an offset term in the models. As in the first set of analyses, we used AIC scores to compare the relative explanatory power of a model that included just the ecological variables to one that also included distance from the nearest road and distance from the nearest human settlement.

#### 2.4.2 Diel Variation in Baboon Activity: How Is Terrestrial Behavior Distributed Across the Diel Cycle, Do These Patterns Differ Between the Two Grids, and to What Extent Do the Two Baboon Populations Show Temporal Partitioning With Their Respective Predator Species?

To explore temporal patterns in baboons' terrestrial activity, we used data from both camera trap grids. First, we investigated when baboons were most often active on the ground during the circadian cycle and whether this differed between grids. For this, we fitted a negative binomial GLMM to the camera trap data to test whether baboon RAI varied significantly by diel period (dawn, day, dusk, or night). We included an interaction term between diel period and location (GNP or FC), based on the hypothesis that baboons' diel activity patterns would be different in a landscape inhabited by lions compared to a landscape inhabited by leopards. Woody cover, distance from water, and localized risk were included as fixed variables, based on results from the spatial models, and camera site was included as a random variable to account for repeated sampling across diel periods. The log‐transformed value of total operating hours per camera per diel period was included as an offset term. All models were fitted using functions from the “lme4” package in R (Bates et al. 2015).

Next, we used temporal overlap analyses to further explore possible differences in the terrestrial activity patterns of GNP and FC baboons, and to test the extent to which baboon activity overlapped with predator activity across each grid. For these analyses, we calculated a coefficient of temporal overlap, d‐hat, for each pair of diel activity distributions (i.e., baboons in GNP vs. baboons in FC, baboons in GNP vs. lions, and baboons in FC vs. leopards), where 0 represents no temporal overlap and 1 represents complete overlap. We used the d‐hat1 formula for sample sizes under 50 independent detections and the d‐hat4 formula for those over 50. We used bootstrapping with 10,000 resamples to estimate 95% confidence intervals for each overlap estimate. These analyses were performed using the “overlap” package in R (Ridout and Linkie 2009). We also used the compareCkern function in the “activity” package in R (Rowcliffe 2022) to estimate the probability that the activity patterns in each pair came from the same distribution. All analyses were conducted in R v4.1.1 (R Core Team, 2021).

### 2.5 Ethical Note

This work was carried out with ethical clearance from Oxford University (APA/1/5/ACER/23Jan2018) and from the Ministry of Tourism and the Gorongosa Restoration Project in Mozambique (permit numbers PNG/DSCi/C114/2018 and PNG/DSCi/C93/2018). All data were collected remotely, and researchers did not come into contact with the animals.

## 3 Results

Table 2 summarizes the camera metadata and image data collected across the GNP and FC grids. There was a broad range of woody cover values across the GNP cameras (mean: 52%, range: 20%–71%) compared to the higher and more homogenous coverage across the FC grid (mean: 67%, range: 58%–78%). On average, GNP cameras were further from water and settlements but closer to roads than FC cameras. Overall, more animal activity was captured across the GNP grid (approximately 3.4 independent detections per operating day) than the FC grid (approximately 0.9 independent detections per operating day). Baboons accounted for 15% of all detections across the GNP grid, compared to only 8% of those across the FC grid. Across the GNP grid, lions accounted for 0.05% of detections. Across the FC grid, leopards accounted for 0.04% of detections. Only two individual leopards were conclusively identified from the data (Easter et al. 2020). No other large carnivores were detected across either grid. Human activity was much higher across the FC grid (18% of detections) than the GNP grid (0.03% of detections).

**TABLE 2:** Summary of camera metadata and image data collected across GNP and FC grids. All detections were classified using a 30‐min independence interval.

	GNP grid	FC grid
Camera metadata: mean (range)		
Woody cover (%)	52 (20–71)	67 (58–78)
Distance from the lake (km)	9 (1.3–19.4)	NA
Distance from the river (km)	3.8 (0.1–8.7)	1.8 (0–5.7)
Distance from the road (km)	0.6 (0–2.3)	2.1 (0–6.3)
Distance from the settlement (km)	10.7 (1.1–20.7)	8.3 (0.5–19.4)
Independent species detections		
Total	89,349	1807
Baboon	13,659 (15%)	147 (8%)
Lion	54 (0.06%)	0 (0%)
Leopard	0 (0%)	8 (0.04%)
Human/vehicle	30 (0.03%)	319 (18%)
Sites with detections of		
Baboon	60 (100%)	56 (75%)
Lion	19 (32%)	0 (0%)
Leopard	0 (0%)	6 (8%)

### 3.1 Spatial and Seasonal Variation in Baboon Activity

#### 3.1.1 GLMM Results From Seasonal Data (GNP Grid Only)

**FIGURE 2:**
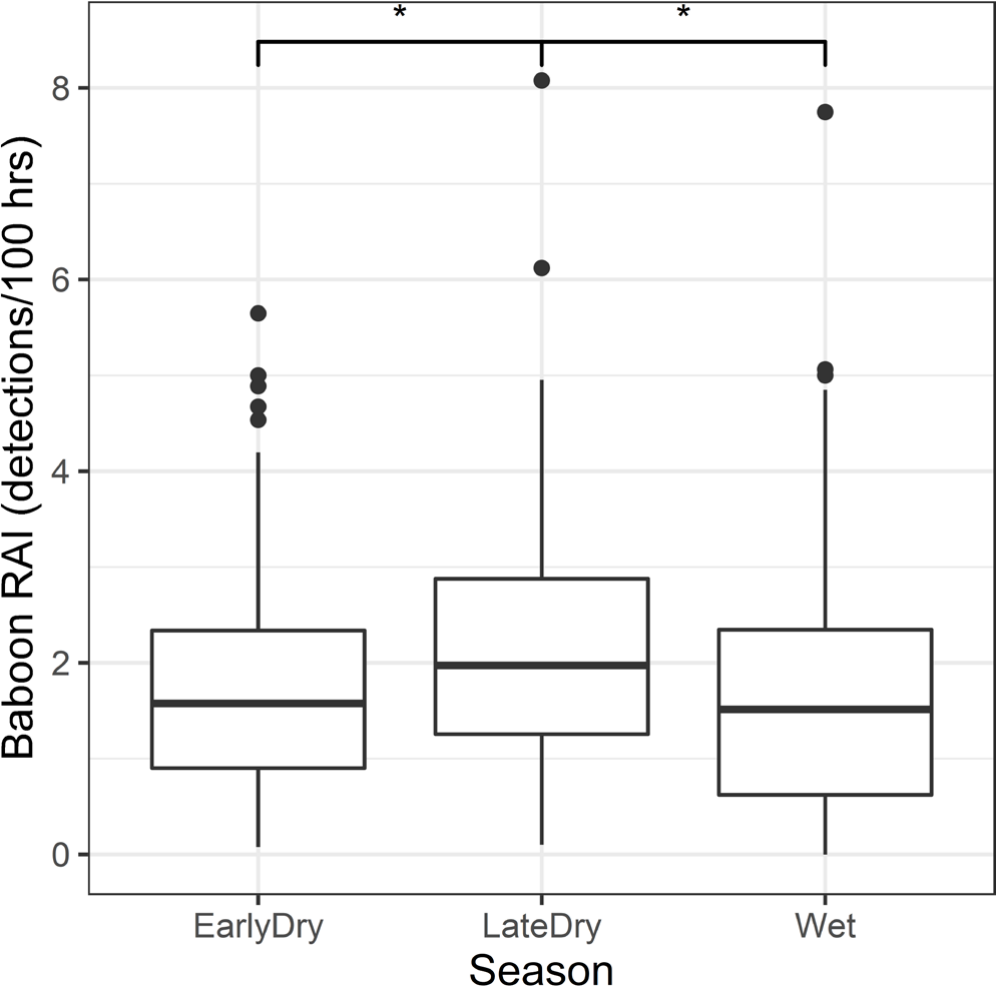
Boxplot showing seasonal differences in the relative activity indices (detections per 100 operating hours) of GNP baboons. Asterisks indicate statistically significant differences between pairs. The line at the center of each box denotes the median value (50th percentile), and each box contains the 25th to 75th percentiles of the data for the given variable. The whiskers extend to the 5th and 95th percentiles, and dots represent values beyond these bounds, considered outliers.

Of the two GLMMs fitted to the seasonal GNP grid data, the model with only ecological and seasonal variables had a lower AIC score (AIC = 2988.6) than the model which also included anthropogenic variables (AIC = 2991.5). We therefore consider it the better model for explaining variation in these data. Table 3 summarizes the associations of each of the model's fixed variables with baboon RAI. These results suggest that baboon terrestrial activity did not vary consistently with woody cover, distance from water, nor the localized presence of predators. There is strong evidence (*p* < 0.001) that across the grid, baboon terrestrial activity was higher in the late dry season than at other times of year. These seasonal differences are shown in Figure 2.

**TABLE 3:** Ecological and seasonal factors associated with baboon activity across the GNP grid. Asterisks indicate statistical significance.

	Estimate	Std. error	*z* value	Pr(>|z|)
(Intercept)	−4.29	0.11	−40.00	< 0.001***
Woody cover	0.00	0.09	0.04	0.97
Lake distance	−0.11	0.10	−1.18	0.24
River distance	0.03	0.09	0.36	0.72
Risk: Predator	0.29	0.22	1.32	0.19
Season: Late dry	0.27	0.02	12.65	< 0.001***
Season: Wet	−0.01	0.03	−0.50	0.62

*** *p* < 0.001.

#### 3.1.2 GLM Results From Data Collected Across Both Grids

**FIGURE 3:**
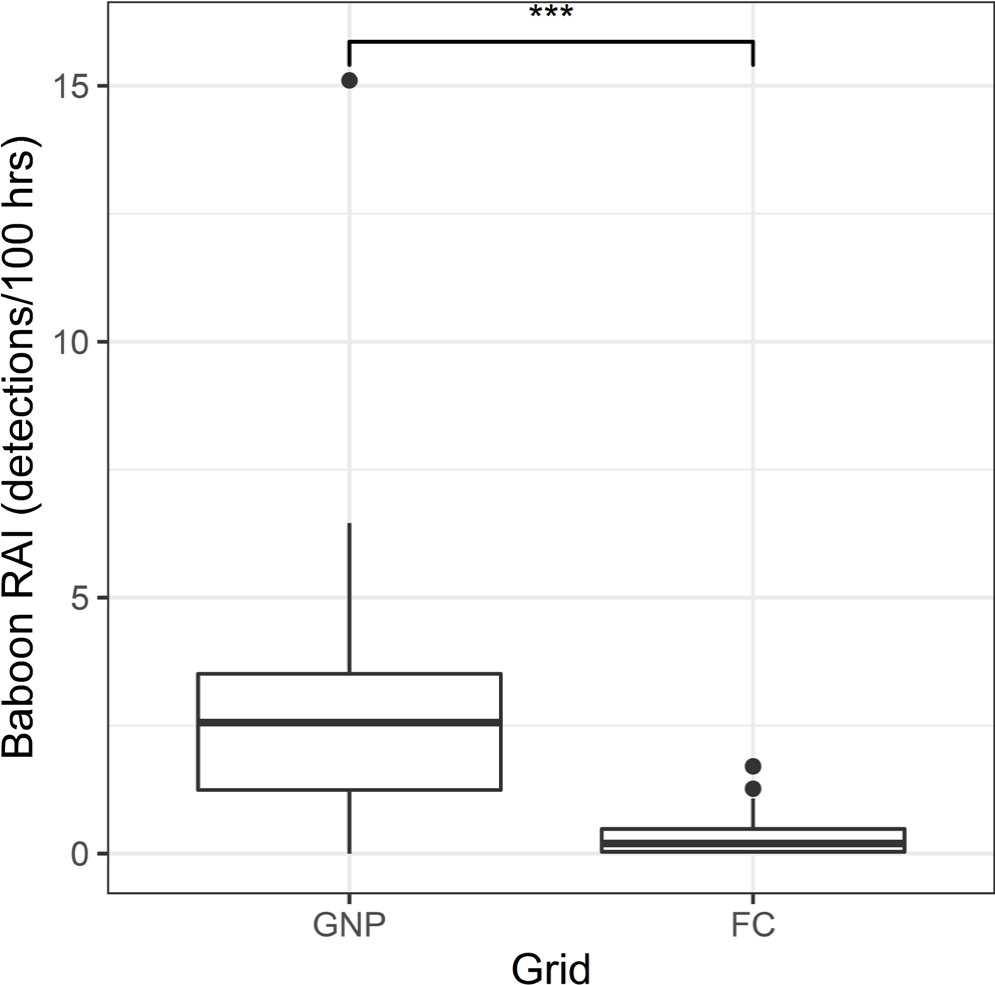
Boxplot showing differences in the relative activity indices (detections per 100 operating hours) of baboons across the GNP and FC grids. Asterisks indicate statistically significant differences between pairs. The line at the center of each box denotes the median value (50th percentile), and each box contains the 25th to 75th percentiles of the data for the given variable. The whiskers extend to the 5th and 95th percentiles, and dots represent values beyond these bounds, considered outliers.

Of the two GLMs fitted to the dry season data collected across both camera trap grids, it was again the model with only ecological variables that had the lower AIC score (807.53) than the model that included anthropogenic variables (811.2). Table 4 summarizes the associations of each of this model's fixed variables with baboon RAI. Again, these results indicate that baboon terrestrial activity did not vary consistently with woody cover, distance from water, or the localized presence of predators. There is strong evidence (*p* < 0.001) that terrestriality was more frequent across the GNP grid than across the FC grid (see Figure 3).

**TABLE 4:** Ecological factors associated with baboon activity across the GNP and FC grids. Asterisks indicate statistical significance.

	Estimate	Std. error	*z* value	Pr(>|z|)
(Intercept)	−5.01	0.55	−9.16	< 0.001***
Woody cover	−0.01	0.01	−1.43	0.15
Water distance	−0.01	0.03	−0.39	0.70
Risk: Predator	−0.11	0.23	−0.50	0.62
Location: GNP	2.11	0.29	7.17	< 0.001***

*** *p* < 0.001.

### 3.2 Diel Variation in Baboon Activity

#### 3.2.1 GLMM Results From Diel Data (Both Grids)

**FIGURE 4:**
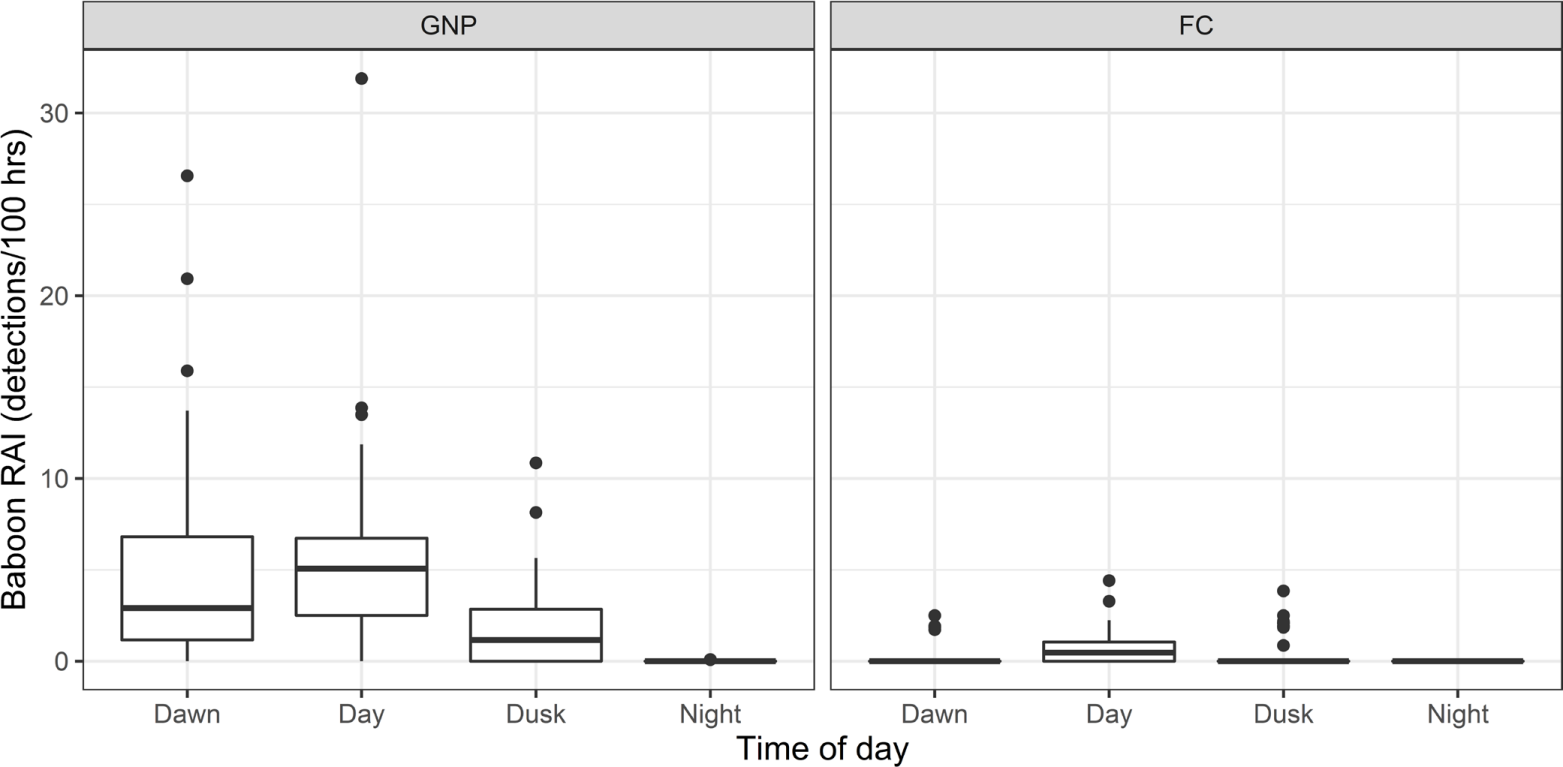
Box plot showing differences in the relative activity indices (detections per 100 operating hours) of baboons across diel periods for each camera trap grid. The line at the center of each box denotes the median value (50th percentile), and each box contains the 25th to 75th percentiles of the data for the given variable. The whiskers extend to the 5th and 95th percentiles, and dots represent values beyond these bounds, considered outliers.

Table 5 summarizes the output from the GLMM that was fitted to diel data from both the GNP and FC grids. There is strong evidence (*p* < 0.001) that baboon activity was higher during the day than during other diel periods, and that there is a statistically significant interaction between diel period and location. The distribution of baboon detections across diel periods for each grid is depicted in Figure 4.

**TABLE 5:** Ecological and diel factors associated with baboon activity across the GNP and FC grids. Asterisks indicate statistical significance.

	Estimate	Std. error	*z* value	Pr(>|z|)
(Intercept)	−6.83	0.50	−13.75	< 0.001***
Woody cover	−0.13	0.10	−1.27	0.21
Water distance	0.13	0.10	1.36	0.17
Risk: Predator	0.10	0.22	0.44	0.66
Location: GNP	3.28	0.54	6.04	< 0.001***
Diel: Day	1.72	0.50	3.45	< 0.001***
Diel: Dusk	0.81	0.58	1.38	0.17
Diel: Night	−19.16	2974.64	−0.01	0.99
GNP*Day	−1.51	0.52	−2.92	0.004**
GNP*Dusk	−1.71	0.61	−2.83	0.005**
GNP*Night	12.46	2974.64	0.00	0.99

** *p* < 0.01.

*** *p* < 0.001.

#### 3.2.2 Coefficients of Overlap

**FIGURE 5:**
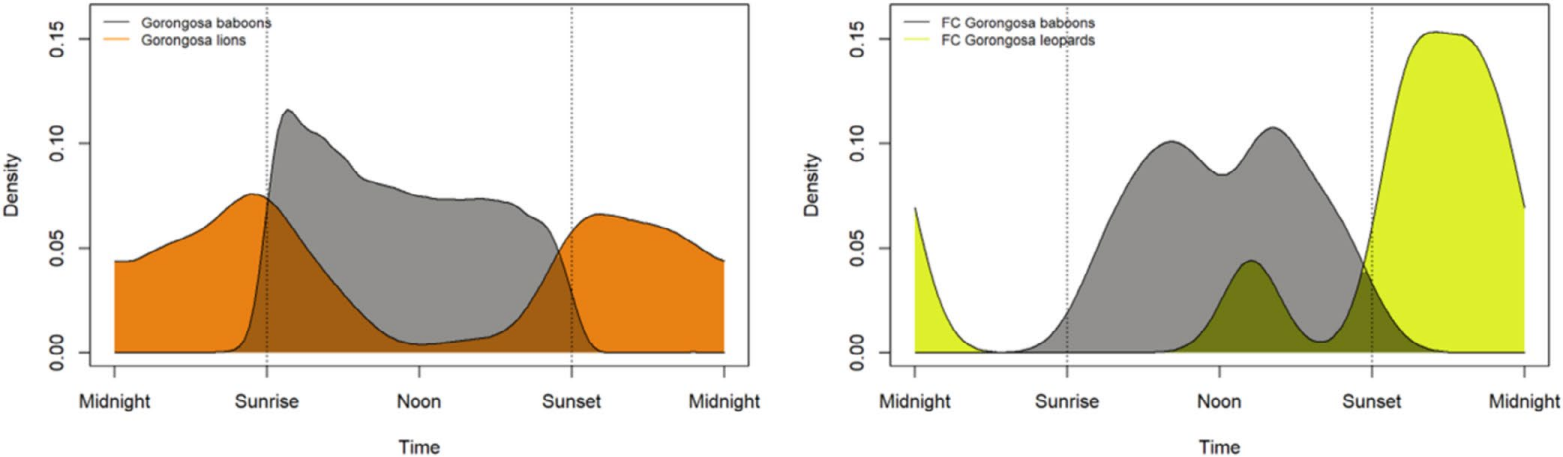
Graphs depicting the temporal overlap between detections of baboons (gray) and apex predators at each site (GNP lions in orange and FC leopards in yellow). Dotted vertical lines represent sunrise and sunset, standardized across sites and seasons. It should be noted that there are too few leopard detections to represent an accurate portrayal of their activity patterns, but we include the results here to show when the eight leopard detections were captured.

Table 6 summarizes the results from the activity overlap analyses between baboons and their predators, and between the GNP and FC baboons. The coefficient of overlap between the GNP and FC baboons is 0.84, likely reflecting the fact that both populations are almost entirely diurnal. While overlap is high, the probability that their activity patterns come from the same distribution is 0.02, suggesting that there are significant differences in how their terrestrial activity was spread across the day. The coefficient of overlap between baboons and lions in GNP was greater than that between baboons and leopards in FC, suggesting that baboons might exhibit greater temporal partitioning with the latter predator species. However, the large confidence interval around the baboon‐leopard d‐hat value highlights that there are too few leopard detections to get a precise portrayal of their activity patterns in the FC area. Figure 5 shows the kernel density distributions for baboon and apex predator detections across the GNP and FC grids, where the area under each curve is equal to 1 and times are scaled to sunrise and sunset.

**TABLE 6:** Coefficients of overlap between baboon populations and between the baboons and predators at each site.

	Overlap coefficient (d‐hat)	Confidence interval	*p*
GNP and FC baboons	0.84	0.79–0.89	0.02*
GNP baboons and lions	0.32	0.21–0.42	< 0.001***
FC baboons and leopards	0.16	0.00–0.42	< 0.001***

*Note:* Confidence intervals were estimated using bootstrapping with 10,000 resamples, and *p*‐values represent the probability that the two activity patterns come from the same distribution.

## 4 Discussion

### 4.1 No Localized Effects of Predator Presence

In this study we used terrestrial camera trap data to explore how terrestriality varies with risk, alongside other ecological factors that might affect baboons' use of the ground. We explored the effects of both spatial variation in predator activity and hypothesized differences in the level of perceived risk across the 24‐h cycle and of different predator species. We first tested whether localized predator presence affected terrestriality across two populations of baboons, hypothesizing that they might avoid, or be more arboreal in, areas where predators are more active. In these analyses we included woody cover, proximity to water, proximity to roads and human settlements, and seasonality as other potential explanatory variables of baboon terrestrial activity. Contrary to our hypothesis, we found no evidence that baboons from these populations were less active in the localized areas where lions or leopards were detected. While this might indicate that they were not spatially avoiding these predators, the results could instead reflect that our measure of predator activity was too coarse to detect fine‐scaled avoidance strategies in the landscape. Baboons might avoid predators in real time as they detect them in certain locations, but our results suggest that in these populations they either did not avoid predator‐occupied areas over longer time scales, or that our methods did not accurately identify the riskiest areas in the landscapes. Future studies could explore some of the nuances that arise from interactions between variables. For example, across the GNP grid, lion activity was more likely to be detected closer to Lake Urema and closer to roads (Gaynor et al. 2021). Further investigation is needed to understand how baboons navigate trade‐offs between this heightened risk and the benefits of their own proximity to water and use of roads, particularly across the seasonal cycle.

### 4.2 Differences in Terrestrial Baboon Activity Across Seasons and Grids

We found no evidence that terrestriality in these populations varied reliably with woody cover, proximity to water, or proximity to anthropogenic features in the environment. Across the GNP camera trap grid, we did find season to be a significant predictor of terrestrial activity, with baboons more active on the ground during the late dry season than at other times of year. In the analysis of data from both grids, terrestrial baboon activity was found to be significantly higher across the GNP grid than the FC grid.

Interpretations of these results must include the consideration that low detection rates of baboons by terrestrial camera traps could indicate that they are not using an area at all, or that they utilize arboreal rather than terrestrial travel in that area and are therefore not captured by cameras close to the ground. For example, the effect of season on baboon activity across the GNP grid has multiple interpretations. Baboon detections might have been lower in the wet season because baboons did not need to move around as much to find food or water, and therefore visited certain areas less frequently. Alternatively—or perhaps as an additional factor—the wet season likely coincides with greater availability of arboreal resources like fruit, while the dry season might require baboons to spend more time on the ground traveling to scarce water sources or foraging for resources like grasses or underground storage organs. As the FC grid was only deployed during the dry season, we could not explore whether FC baboons showed the same seasonal shift in activity patterns as that seen in GNP baboons.

We found strong evidence that terrestrial behavior across the GNP grid was more frequent than across the FC grid. The size of this difference suggests that it likely captures several effects that cannot be untangled with the available data. As seen in Table 2, the percentage of woody cover was higher and more homogeneous across the FC grid than the GNP grid. It might seem parsimonious that this indicates greater tree coverage and that baboons living in an area with more trees simply spend less time on the ground. However, our results indicate that woody cover was not a significant predictor of baboon activity (despite a range of 20%–78% coverage across both grids), perhaps indicating that vegetation was not the primary driver of their substrate use. Unfortunately, our woody cover metric does not distinguish between trees and shrubs and therefore might mask an effect of tree availability.

Another key reason why baboons might have been detected at higher rates across the GNP grid than the FC grid is population size. An estimate from aerial surveys suggests that approximately 60 troops of baboons range across the GNP grid, but it is not clear how many troops live in the FC area, nor do we have an estimate of average troop sizes in either area. It is likely that a higher density of baboons in the GNP area accounted for higher detection rates across this grid. Additionally, the presence of different apex predators across the grids might have been a contributing factor to different patterns in terrestrial activity. As previously noted, leopards are thought to be baboons' top predator, while lions generally target larger animals. If the predation rate of baboons is higher by leopards in FC than by lions in Gorongosa, this might contribute to a lower population density of baboons across the FC grid. Furthermore, if baboons *perceive* a higher threat from leopards than they do from lions, they might have been avoiding the FC area and/or spending less time on the ground there to avoid terrestrial encounters with leopards.

Because we do not have estimates of the different population densities and predation rates across the two grids, it is valuable to look at other more comparable patterns in the terrestriality of the two populations to determine how they responded to different apex predators in their respective environments. In our second set of analyses, we explored variation in baboon activity over the 24‐h cycle, how this pattern differed across the two grids, and the extent to which the baboons demonstrated temporal partitioning with apex predators in their environments.

### 4.3 Differences in Diel Patterns of Terrestrial Activity Across Baboon Populations

First, we found that baboons across both grids were clearly diurnal, as can be seen in Figure 5. This is reflected in the fact that significantly more baboon detections were recorded during the day than during crepuscular and nocturnal periods, and that there was an 84% overlap in the patterns of terrestrial baboon activity detected across each grid. Despite this high overlap, we found a low probability (0.02) that the two baboon activity patterns came from the same underlying distribution, which indicates that the two baboon populations have distinct activity patterns within the diurnality observed across each grid. Indeed, the distribution of detections across diel periods demonstrates that in FC, baboon terrestriality was more frequent during the day (0.72 ± 0.81 independent detections/100 h) compared to dawn (0.11 ± 0.46) and dusk (0.28 ± 0.77), and no terrestrial baboon detections (*n* = 0) were captured at night. During the comparable months in GNP, baboon activity was high during both dawn (4.77 ± 5.47) and day (5.53 ± 5.00) periods, only declining at dusk (1.74 ± 2.19), with even a few (*n* = 20) detections of baboons on the ground at night (0.01 ± 0.09). Figure 4 provides a summary of these results, while Figure 5 visualizes the difference in baboon activity patterns between grids, clearly showing a peak in terrestrial activity detection soon after sunrise in GNP, while the FC distribution is centered more around the middle of the day.

Our results indicate that the two baboon populations had significantly different diel patterns in their terrestrial activity, and we postulate that this might be due to the presence of different apex predators in each landscape. Figure 5 demonstrates that the apex predators across both habitats were predominantly nocturnal and crepuscular, and that baboons showed significant temporal partitioning with both lions and leopards in their respective environments. Our results indicate that baboons exhibited greater temporal partitioning with leopards in FC than with lions in GNP. However, this result comes with the caveat that the number of leopard detections across the FC grid was low, making it difficult to get a precise indication of their activity patterns.

Despite the small sample, the detections confirm that leopards inhabit the landscape, and that their activity patterns align with the predominantly nocturnal and crepuscular patterns documented at other sites (Havmøller et al. 2020; Searle et al. 2021), although there is some indication of diurnal activity, which has also been documented elsewhere (Isbell et al. 2018). Our results show that the baboons sharing their environment with leopards in FC confined most of their terrestrial activity to the middle of the day, which is similar to patterns documented at other sites inhabited by both baboons and leopards (Havmøller et al. 2020). A small dip in baboon activity around midday does align with the detection of diurnal leopard activity, but its significance is difficult to interpret with such a small sample of leopard detections. Our statistical comparison of baboon activity patterns across FC and GNP indicates that GNP baboons utilized the ground from very early in the morning and even sometimes at night, despite this activity overlapping with lions' active periods. This perhaps indicates that the GNP baboons perceived less risk in an environment inhabited by lions than the risk perceived by FC baboons living alongside leopards, or perhaps that there were other factors driving the GNP baboons to use the ground during risky crepuscular hours.

### 4.4 Future Research and Implications

There are two clear research avenues to explore these diel patterns further. First, Gorongosa provides an ideal setting for a longitudinal natural experiment. While this study documents baboon activity in an environment without leopards and with minimal predation risk from the few lions in the park, Gorongosa baboons' landscape of fear is likely changing. Since 2018, two packs of wild dogs have been released and have successfully reproduced within the park, and six leopards have been reintroduced to the area. Ongoing camera trap data collection will thus provide valuable insights into how baboon terrestriality and diel activity patterns change with increasing risk. Such data, combined with in situ research, will also be useful for exploring what drives terrestrial activity in theoretically “risker” diel periods. For example, our study suggests that GNP baboons particularly utilize the dawn period, perhaps driven by the benefits of getting up earlier to forage in the cooler hours of the day, or by competition across troops to reach resources earlier in the day. Our study also documented a few cases of nocturnal activity in GNP baboons. Further camera trap and collaring data could be used to investigate whether this correlates with lunar cycles, resource availability, or other factors. Another avenue for research involves cross‐site comparisons to further examine how particular predator species and/or the carnivore diversity within different landscapes affects baboon activity patterns.

Although terrestrial behavior is only documented in a few primate species, it correlates with a variety of morphological and behavioral traits (e.g., larger body sizes, group sizes, and home ranges, as well as longer day ranges (Clutton‐Brock and Harvey 1977)) that facilitate easier and broader dispersal across a range of terrains, resulting in the occupation of a greater variety of habitat types by terrestrial primates (Galán‐Acedo et al. 2019). Furthermore, studies have found that even a partly terrestrial lifestyle has a positive relationship with tool use and manipulation complexity in primates, as well as birds (Bandini et al. 2022; Falótico and Ottoni 2023; Heldstab et al. 2016; Meulman et al. 2012; Ottoni and Izar 2008; Wright et al. 2019). We see many of these traits in humans—the most terrestrial of primates—and our hominin lineage is largely defined by a shift in locomotor style to obligate terrestrial bipedalism. The origins and drivers of primate terrestriality are therefore of key interest for understanding human behavioral evolution.

This study highlights the potential impact of variation in risk on primate terrestriality at a landscape scale, with a particular emphasis on temporal dynamics and predator species. To build a fuller picture of the effects of dynamic landscapes of fear on primate behavior, it will be necessary to tease apart the interactions between many spatial and temporal variables. For instance, while we find no effect of woody cover on baboon terrestriality in this study, it is likely that a more precise measure of tree coverage might reveal thresholds above and below which baboons' terrestrial patterns are drastically altered. And while each of the landscapes included in this research was only inhabited by one apex predator, ecosystems with several predators will have more complex dynamics of spatial and temporal partitioning among members of the guild, with different consequences for prey behavior. Remote‐sensing methodologies are improving our abilities to monitor community ecology at broad scales and with unprecedented scope for longitudinal and simultaneous monitoring of species. These technologies, combined with more traditional in situ observational research, will facilitate more accurate and nuanced models of how risk impacts behavioral ecology and evolution in primates and other taxa.

## 5 Conclusion

Primate terrestriality is not common and most species have constrained diel activity patterns. Predation pressure is often implicated in the evolution of such behaviors, but it has been hard to study—particularly at broad scales and before the advent of remote‐sensing technologies. This study investigates how the terrestrial activity patterns of baboons are affected by risk, as well as ecological variables and the seasonal and diel cycles. We found no effects of localized spatial variation in predator presence, nor woody cover, water availability, or anthropogenic variables on baboon activity across two camera trap grids. We did find that GNP baboons were more active on the ground in the late dry season and that use of the ground differed between the two populations studied here. These differences might be attributable to the presence of leopards in one of the landscapes limiting baboon terrestriality to “safer” periods of the day. While further research is needed, these results highlight how different predator species might contribute differently to baboons' landscapes of fear, and might have a greater impact on baboons' temporal rather than spatial partitioning behaviors. These insights contribute to our growing understanding of complex community ecology dynamics as well as the emergence and evolution of primate behaviors.

## Author Contributions

**Philippa Hammond:** conceptualization (lead), data curation (equal), formal analysis (equal), funding acquisition (equal), investigation (lead), methodology (equal), project administration (lead), resources (supporting), visualization (equal), writing – original draft (lead). **Kaitlyn Gaynor:** conceptualization (supporting), data curation (equal), formal analysis (equal), funding acquisition (lead), methodology (equal), supervision (supporting), validation (supporting), visualization (equal), writing – review and editing (supporting). **Tara Easter:** conceptualization (supporting), data curation (equal), formal analysis (supporting), methodology (equal), resources (equal), validation (supporting), writing – review and editing (supporting). **Dora Biro:** conceptualization (supporting), formal analysis (supporting), methodology (supporting), supervision (lead), writing – review and editing (supporting). **Susana Carvalho:** conceptualization (supporting), formal analysis (supporting), funding acquisition (supporting), investigation (supporting), methodology (supporting), supervision (lead), writing – review and editing (supporting).

## Conflicts of Interest

The authors declare no conflicts of interest.

## Data Availability

The data collected and analyzed for this study are available from the corresponding author upon reasonable request.
